# Study of Structure and Phase Formation During Thermal Treatment of Geopolymer Compositions Based on Mineral Waste

**DOI:** 10.3390/ma18174132

**Published:** 2025-09-03

**Authors:** Elena A. Yatsenko, Sergei V. Trofimov, Yuri V. Novikov, Boris M. Goltsman, Vitaliy V. Sergeev

**Affiliations:** 1 Faculty of Technology, Platov South-Russian State Polytechnic University (NPI), Prosveshcheniya Street, 132, Novocherkassk 346428, Russia; 2 Peter the Great Saint Petersburg Polytechnic University, Politekhnicheskaya Street, 29, Saint Petersburg 195251, Russia

**Keywords:** steel slag, drilling sludge, industrial waste, porous geopolymers, thermal transformation, foaming, structure, thermal stability

## Abstract

A comprehensive study was conducted to investigate the influence of mineral waste on the thermal stability of foamed geopolymer materials. The study’s objects were steelmaking slag (SS) from the Taganrog Metallurgical Plant, drilling sludge (DS) from the Sutorminskoye oil field, and an ash and slag mixture (ASM) from the Novocherkasskaya SDPP. The utilisation of drilling sludge as an additive in the production of geopolymers has been proposed for the first time. The study involved the development of alkaline activators based on solutions of sodium and potassium silicates and their hydroxides. The samples were synthesised with varying proportions of steelmaking slag and drilling sludge, and physicochemical, mechanical and high-temperature studies were conducted to ascertain the optimal composition. X-ray phase analysis of the synthesised samples was conducted. An investigation was conducted into alterations in the phase composition of the material as a consequence of heat treatment. Proposals were hereby made for the mechanisms of the formation of new phases. The study identified an alkaline activator based on a solution of silicate and sodium hydroxide, with the introduction of 10% steelmaking slag into the component mixture, as the most effective mixture. The resultant geopolymers exhibited a density of 311 kg/m^3^ and an ultimate compressive strength of 1.54 MPa.

## 1. Introduction

Currently, the world community is acutely aware of environmental changes that manifest themselves in environmental pollution and its impact on health. As is known, in conditions of rapid economic development, the accumulation of industrial solid waste (ISW) is one of the greatest environmental problems [1,2]. Waste includes various industrial wastes, such as in metallurgy—steelmaking slags, coke, and metal moulding residues; in oil production and mining—drilling sludge and waste rock; in energy—fuel slag, fly ash, or a mixture of them.

According to various estimates, in the energy sector alone, 750 to 900 million tonnes of ash and slag waste are generated annually, making them the highest-tonnage wastes. For example, China produces 580 million tonnes of ash and slag waste annually, India produces 220 million tonnes, the European Union produces 100 million tonnes, the USA produces 45 million tonnes, and Russia produces about 22 million tonnes [3,4,5,6,7]. Steel slag is also characterised by similarly high rates of formation: approximately 18 million tons are produced each year in the European Union, 62 million tons are produced in India, 100 million tons are produced in China, approximately 10 million tons are produced in the USA, and the same amount is produced in Russia [8,9,10,11,12,13].

In turn, the volume of drilling waste is directly related to annual oil production. According to a report from the US Energy Information Administration (EIA), global production reached 81,998 Mb/d (million barrels per day) in 2023 alone [14]. However, there is no precise data on the amount of drilling waste generated. It is estimated that up to 0.5 m^3^ of waste is generated for every metre drilled, totalling between 1000 and 5000 tonnes per well [15]. The main indicators of soil contamination by drilling waste are high concentrations of Sr, Ba, petroleum hydrocarbons, and Cl^−^. For example, Anatoly Opekunov et al. examined the chloride ion content in the vicinity of waste accumulation. Their findings revealed that the concentration of chloride ions in this area was found to be 25 times higher than in areas situated 200 m away from the site of waste accumulation [16]. The presence of listed harmful components in the environment can have a significant impact on agricultural areas, with the potential of contaminating soil, seeping into rivers, lakes, or groundwater, and causing considerable damage to the environment [17]. In addition to the necessity of recycling waste in order to preserve the ecosystem, the subject of listed waste has attracted the attention of many researchers as a potentially green and sustainable building material due to its unique chemical composition, physical qualities, and mechanical properties [18,19,20].

In recent years, geopolymers have emerged as a particularly promising method for the utilisation of aluminosilicate waste. Geopolymers are a novel class of innovative building materials. These are air binders that are obtained by means of alkaline activation of aluminosilicate components in raw materials of varying provenance, including natural, artificial, and industrial sources [21,22,23,24,25,26]. Depending on the required application, a distinction is made between geopolymer concrete and porous geopolymer materials. Geopolymer concrete is a more economical alternative to Portland cement due to its favourable physical and mechanical properties and the use of relatively abundant man-made raw materials. This is achieved by partially replacing clinker with industrial by-products [27,28,29,30]. It is clear that Portland cement production is associated with the formation of large amounts of CO_2_, significant energy consumption, and the use of scarce natural raw materials. Geopolymer production does not have these disadvantages because it does not require high temperatures since the waste products have already undergone the firing stage. Reducing CO_2_ emissions and making significant energy savings is as easy as using low-temperature geopolymer synthesis regimes. Geopolymers’ molecular structures allow heavy metal ions to be incorporated, greatly reducing their toxic effect. Consequently, porous geopolymers find wide application in functional materials, such as adsorbents for the removal of metal ions [31], wastewater treatment materials [32,33,34], catalysts [35], and, in the future, could be used as biomaterials for bone healing [36].

Nonetheless, the most pervasive potential utilisation of porous geopolymers is as a structural material within the construction industry, such as in the form of thermal insulation within road and civil engineering applications [37,38,39]. It is established that the optimal thermal insulation materials should exhibit low density and thermal conductivity coefficient [40]. Moreover, it is imperative to acknowledge the significance of heat resistance, which is defined as the capacity of the material to maintain its functional characteristics when subjected to elevated temperatures without undergoing melting. Evidence of the significance of this property in the manufacturing of thermal insulation materials is provided by numerous publications [41,42,43].

As previously stated, the generation of steelmaking slag is currently occurring at relatively high volumes. In order to minimise the detrimental effect on the ecosystem, steelmaking slag is most frequently utilised as an aggregate in the production of concrete, and, to a lesser extent, in the manufacture of bricks, asphalt, and ceramics [9,44,45,46,47,48]. The chemical and phase composition of slag is known to be contingent on the feedstock and the material produced. In general terms, the main oxides present are silicon dioxide (SiO_2_) and calcium oxide (CaO), with iron (Fe_2_O_3_) being present to a lesser extent. This distinguishes the material as a promising modifying additive to aluminosilicate feedstock for the synthesis of porous geopolymers [9,47,48]. The utilisation of steelmaking slag as the primary raw material poses a significant challenge in the synthesis of geopolymers, attributable to the high content of free calcium oxide in the slag. This has been shown to increase the curing rate of porous geopolymers, which has a detrimental effect on their physical and mechanical properties. However, a study has been conducted in which the utilisation of steelmaking slag was 72 wt.% [49].

A significant corpus of research has now been published on the utilisation of steelmaking slag in the production of geopolymer concrete [50,51,52,53]. However, its application in the synthesis of porous geopolymers is less extensive. Moreover, this factor may also be associated with the observation that the volume of publications on porous geopolymers is comparatively lower than the volume of publications on geopolymer concretes. The rationale behind this phenomenon is that geopolymers are a comparatively recent material in the field of geopolymer concretes. Thus, Sang Mingming et al. [49] synthesised a porous geopolymer based on steelmaking slag and sodium metasilicate to remove Cu^2+^, but only its compressive strength of 0.35 MPa was known. Tsaousi G. M. et al. [54] used copper slag and potassium alkaline activator to produce geopolymer foam with a density of 805 kg/m^3^ and a compressive strength of 1.28 MPa. In the study conducted by Guangwei Liang et al. [55], a mixture of blast furnace slag, rice husk ash, and sodium metasilicate was subjected to a compressive strength test, yielding a result of 10.5 MPa at a density of 827 kg/m^3^.

A review of articles on the use of steelmaking slag and drilling sludge in porous geopolymers found no studies that considered the combined effect of liquid glasses of different compositions and the introduction of slag as a modifying additive. It is on this basis that the authors set themselves the task of studying the joint influence on the formation of the structure and physicochemical properties of porous geopolymers. The objective of this study is to contribute to the research field of porous geopolymers, which are regarded as promising materials for the utilisation of waste from the solid fuel power, steel, and oil industries. The fundamental novelty of this work lies in the collaborative investigation of the impact of steel slag and drilling sludge on the thermal properties of porous geopolymer materials, in addition to the examination of their mechanical properties and phase composition. Furthermore, the authors identified a paucity of studies examining the utilisation of drilling sludge in the fabrication of geopolymers, and no studies that have evaluated its impact on the properties of the target material. In this regard, research is being conducted on the influence of drilling sludge on geopolymer materials for the first time. It is important to note that research on the utilisation of steel slag in the fabrication of foamed geopolymers is extremely limited, despite the extensive body of research dedicated to the use of steel slag in the production of dense geopolymer concretes.

## 2. Materials and Methods

### 2.1. Characteristics of Raw Materials

**Figure 1:**
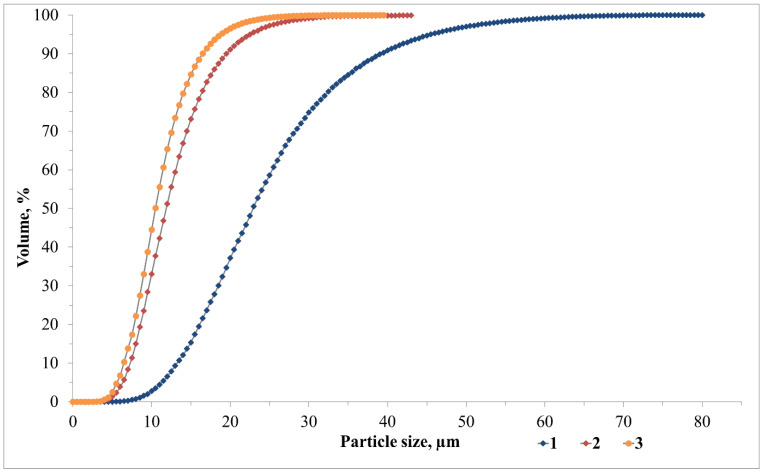
Waste particle size distribution: 1—ASM; 2—SS; 3—DS.

An ash and slag mixture from Novocherkasskaya SDPP (ASM) (Novocherkassk, Russia) was utilised as the primary aluminosilicate raw material in the production of foamed geopolymer materials, with steel slag serving as the secondary raw material. Modifying additives were sourced from the Taganrog Metallurgical Plant JSC (Taganrog, Russia), and drilling sludge was sourced from the Sutorminskoye oil and gas field (Yamalo-Nenets Autonomous Okrug, Russia). The chemical composition of the waste is presented in Table 1. The particle size distributions of the waste materials utilised were obtained by means of a laser analyser designated ‘LASKA-TD’ (BioMedSystem, St. Petersburg, Russia) and are presented in Figure 1. The physical parameters of the waste, including the true density (kg/m^3^), mean particle diameter (µm), and specific surface area (m^2^/kg), are presented in Table 2.

As demonstrated in Table 1, the chemical composition of ASM is dominated by SiO_2_, with substantial quantities of Al_2_O_3_ and Fe_2_O_3_. This factor indicates that the technogenic waste under consideration belongs to the class of aluminosilicate waste, characterised by a high content of iron oxide. Utilising this information, the next step is to consider its potential for use in the production of geopolymeric materials based on ferrosilicate compounds of complex composition. The following structure is proposed for these materials, as illustrated in Equation (1):

–(Ca, Me)–(Fe–O)–(Si–O–Al–O–)–(1)

where Me is an atom of an element of group 1 of the periodic table of chemical elements (Li, Na, K, etc.).

The mechanism of formation of geopolymer chains utilising this particular raw material is primarily driven by the capacity of alkalis to interact with oxides of silicon, aluminium, and iron. This interaction results in the formation of corresponding silicates, aluminates, and ferrites, thereby leading to the establishment of complex inorganic polymer chains.

As is well established, steel slag is formed in significant quantities as a by-product of the steelmaking process using an electric arc furnace. The production of steelmaking slag of varying compositions is achieved through the utilisation of three distinct types of furnaces: oxygen converter slag, electric arc furnace slag and ladle furnace slag [56]. As demonstrated in Table 1, the chemical composition of SS is characterised by a significant presence of CaO, which is indicative of the presence of slag resulting from the smelting of iron and steel. This factor gives the waste a high reactivity due to a certain pozzolanic activity of CaO and some of its compounds, which are capable of forming calcium silicates when the mixture is mixed with aqueous solutions of alkaline activators. Theoretically, this process can be delineated by the derived cascade of Equations (2) and (3):

CaO + H_2_O = Ca(OH)_2_ (2)

nCa(OH)_2_ + (SiO_2_)_y_·(Me_2_O)_x_ = (CaO)_n_·(SiO_2_)_y_ + 2xMeOH (3)

where Me is an atom of an element of group 1 of the periodic table of chemical elements (Li, Na, K, etc.).

However, the presence of excessive amounts of CaO has been demonstrated to exert a detrimental effect on geopolymerisation reactions and geopolymer chain formation. This phenomenon is predominantly attributable to the formation of excessively large quantities of calcium silicates, which consequently engenders a substantial decline in the concentration of reactive OH^−^- and SiO_3_^2−^- ions within the mixture. In addition, this factor leads to a deterioration of the stackability of the mixture as well as to its rapid setting. Furthermore, it has been established that in the production of geopolymers, fly ash is frequently utilised, exhibiting characteristics analogous to metallurgical slags. According to the American Society for Testing and Materials, low-calcium class F fly ash, containing less than 10% free CaO, is preferable to high-calcium class C fly ash, containing more than 10% free CaO. It is hypothesised that fly ash, which is characterised by its high calcium oxide content, impedes polymerisation reactions, compromises mixability, and affects micro-structural characteristics. It is generally accepted that these characteristics are also applicable to steelmaking slag, due to the similarity of the mechanism of their interaction with alkaline activators. Thus, the studied SS can be used in the production of geopolymers only as a modifying additive, due to the low content of silicon and aluminium oxides and the high content of CaO.

In addition, the value of LOI in the chemical composition of SS has a negative value. This factor is indicative of the presence of metallophase in the composition of the waste, which undergoes oxidation during calcination with the formation of the corresponding metal oxides, resulting in an increase in its weight. Table 3 provides a synopsis of the metallophase composition within SS. As demonstrated in Table 3, the predominant constituent of the metallophase is iron, and the specimen exhibits a high degree of magnetic properties. This facilitates the extraction of the metallophase from the slag through the utilisation of magnetic separation techniques.

DS contain 58% silicon and aluminium oxide. Furthermore, it has been observed to contain greater than 5% of potassium oxides, calcium oxides and chloride ions. The latter may have a deleterious effect on the experience of using synthesised drilling-sludge-based geopolymers with metallic structures. The presence of chloride ions in slurries is associated with two factors: the use of a plugging mortar or mining in areas where chloride saline soil is present. Cl^−^ is known to increase the corrosion rate by destroying the passivation film on the steel surface [57]. It is imperative to reduce the number of chloride ions in the sludge in order to obtain optimum physical and mechanical properties in the synthesis of geopolymer materials. This can be achieved by washing the sludge.

**Figure 2:**
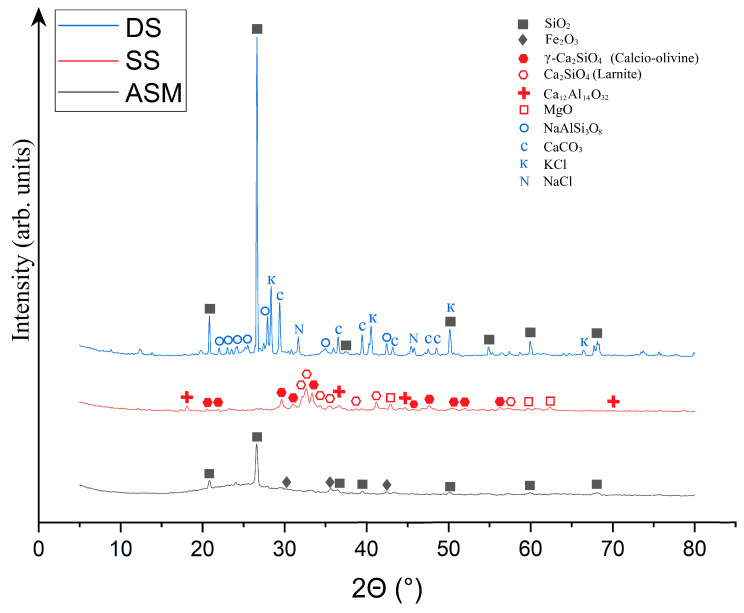
X-ray phase analysis of samples ASM, SS and DS: ∎—SiO_2_; ◆—Fe_2_O_3_; ⬢—γ-Ca_2_SiO_4_; ⬡—Ca_2_SiO_3_; ✚—Ca_12_Al_14_O_32_; **□**—MgO; ⚪—NaAlSi_3_O_8_; C—CaCO_3_; K—Sylvite KCl; N—NaCl.

The ASM, SS, and DS radiographs are shown in Figure 2.

As can be seen from Figure 2, the phase composition of ASM is represented by two crystalline phases: SiO_2_ in the form of quartz, and Fe_2_O_3_ in the form of hematite. This is generally confirmed by the chemical composition of ASM (Table 1), where SiO_2_ and Al_2_O_3_ are some of the main components. In addition, the ASM composition shows a halo in the range of 14–34°, indicating the presence of a glassy amorphous phase in the waste, formed during the melting of the mineral ore portion of the coal fuel. The quantitative composition of ASM is comprised of 88.5 ± 2.3% amorphous and 11.5 ± 0.8% crystalline structure (10.2 ± 0.7% SiO_2_ and 1.3 ± 0.1% Fe_2_O_3_).

The phase composition of SS, as shown in Figure 2, is expressed by the presence of two different forms of calcium orthosilicate–γ-Ca_2_SiO_4_ expressed by the mineral calcic olivine, and Ca_2_SiO_4_ is expressed by the mineral larnite. In general, metallurgical slags are characterised by the presence of these phases. These phases are formed in slag during the high-temperature treatment of primary metal ores, resulting in the interaction of calcium oxides and other calcium-containing constituents with silica from natural minerals or other additives. The presence of the Ca_12_Al_14_O_32_ phase is also observed in SS, which is a calcium aluminate formed during the high-temperature treatment of metallic ores as a result of the interaction of calcium and aluminium oxides. The presence of calcium aluminate testifies to the reactivity of SS because, when mixed with water, it tends to form calcium hydroaluminates capable of interacting with the alkaline components of the activator to form polysialate chains. The crystalline phase represented by MgO in the form of periclase, whose source is the magnesium compounds in the original metal ores, is the least abundant in SS. The quantitative composition of SS is comprised of 92.2 ± 3.3% amorphous and 7.8 ± 0.6% crystalline structure (3.1 ± 0.2% γ-Ca_2_SiO_4_; 2.7 ± 0.2% Ca_2_SiO_3_; 1.2 ± 0.1% Ca_12_Al_14_O_32_; 0.8 ± 0.1% MgO).

The phase composition of drilling sludge is expressed by the presence of ordered albite (NaAlSi_3_O_8_), calcite (CaCO_3_), β-quartz (SiO_2_), sylvin (KCl), and halite (NaCl) phases. β-Quartz and calcite are components of the rock extracted during well development. Silvin is present as an impurity in drilling sludge, the source of which is plugging fluids containing solutions of this salt. Halite is likely to be a component of the groundwater and formation water trapped in the drilling sludge during well development, and may also be a component of the plugging mud. DS is represented by a crystalline structure that contains 48.4% ± 0.7% SiO_2_, 26.9% ± 0.8% NaAlSi_3_O_8_, 18.8% ± 0.7% CaCO_3_, 1.3% ± 0.1% Sylvite KCl, and 4.6% ± 0.4% NaCl.

For the preparation of the alkaline activator solution, we used alkalis with the content of a 99% basic substance: granulated sodium hydroxide NaOH (SANTRADE, Lermontov, Russia) and potassium hydroxide KOH (LenReactiv, Saint-Petersburg, Russia). A 55% aqueous solution of sodium metasilicate Na_2_O(SiO_2_)_n_ (silicate modulus 2.94) and a 52% aqueous solution of potassium metasilicate K_2_O(SiO_2_)_n_ (silicate modulus 3.08) were used as binder components. The listed metasilicates were produced by LLC «Maria-Trade», Ekaterinburg, Russia. Spherical dispersed aluminium (powder) of ASD-1 grade with a purity of 99% and a specific surface area of 152 m^2^/g (GK Metal Energo Holding, Yekaterinburg, Russia) was used as the foaming agent.

### 2.2. Methods

Qualitative X-ray phase analysis of the synthesised porous geopolymers was performed on an ARLX’TRA diffractometer (Thermo Fisher Scientific, Waltham, MA, USA) with reflection beam focusing using the Bragg–Brentano method. The detailed methodology of thermal conductivity determination is presented in the authors’ previous studies. A semi-quantitative analysis was conducted using the materials analysis using diffraction (MAUD) software (v2.9993, build 532, Trento, Italy), which is based on the Rietveld method. The obtained X-ray diffraction patterns were then optimised using the built-in least-squares algorithm, after which the phases’ concentrations were determined.

The thermal conductivity of geopolymers was measured using a thermal conductivity meter (ITP-MG4“100/Zond”, SKB StroyPribor, Chelyabinsk, Russia) by the steady-state heat flow method. The detailed methodology of thermal conductivity determination is presented in the authors’ previous studies [25].

The methodology and equipment required to determine the linear dimensions and some physical and chemical properties (volume, density, porosity, ultimate compressive strength) of the synthesised porous geopolymers are described in the authors’ previous studies [26,58]. Each recorded value consists of five test repetitions. The calculation of the standard error was achieved by determining the standard deviation of the data sample, utilising Equation (4):

(4) $$Z=\surd (\sum (x-\bar{x}{)}^{2}/n-1)$$

where x is property value obtained in the experiment; $\bar{x}$—average value of experimental properties; n—the selection of experimental data from 5 repetitions.

A portable thermal imager (CEM DT-9897H, Shenzhen Everbest Machinery Industry CO, Shenzhen, China) was used to determine the surface temperature of the geopolymer inside the chamber furnace. Temperature is determined by measuring the amount of infrared energy emitted from the surface of the sample. The temperature measurement range is from −20 °C to +1500 °C with an accuracy of ±2 °C (but not more than 2%). The thermal sensitivity of the instrument (NETD) at 30 °C is 0.05 °C. Infrared images were analysed using Thermview Pro software (v2.0.9, Shenzhen Everbest Machinery Industry Co, Nanshan, Shenzhen, China).

An electric chamber furnace, TK.8.1300.N.1F (LLC ‘Thermoceramics’, Moscow, Russia), was used to determine the melting temperature of the optimum compositions. The synthesised samples were heated to a temperature of 1300 °C at a rate of 10 °C/min.

### 2.3. Synthesis of Porous Geopolymer Materials

**Figure 3:**
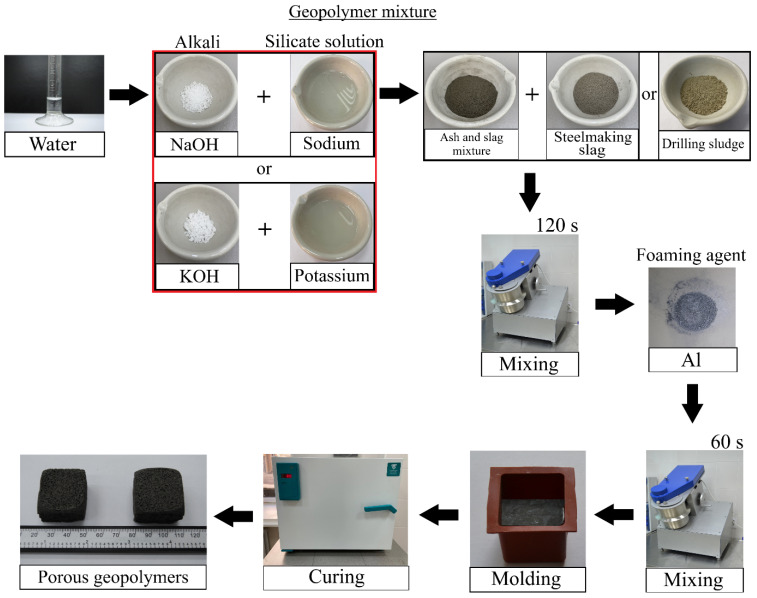
Technology of synthesis of porous geopolymer materials.

As mentioned above, ASM, SS and DS were used as precursors; spherical dispersed aluminium was used as a foaming agent. The following labelling was used to indicate the compositions: the coefficient before the composition denotes the waste «A»—ash and slag mixture—and then their mass content in the mixture «75, 70, 65, 60 and 55»; «S»—steelmaking slag—«D»—drilling sludge with mass contents «0, 5, 10, 15 and 20». «Na» indicates the use of sodium hydroxide and sodium metasilicate to prepare the alkaline activator, «K» indicates the use of potassium hydroxide and potassium metasilicate, respectively. The final composition of A60S15K consists of: 60 wt.% ash and slag mixture, 15 wt.% steelmaking slag, and potassium hydroxide and potassium metasilicate were used as alkaline activator. The technological scheme of the synthesis of porous geopolymers is shown in Figure 3, and the composition of the raw material mixture is shown in Table 4.

For the preparation of the alkaline solution, a separate container was used in which a pre-measured alkali suspension was dissolved in distilled water at room temperature 20 ± 1 °C to obtain a molar concentration of 12 mol/L. This molar concentration is the optimal concentration according to previous studies by the authors [25,59]. The prepared alkaline solution was mixed with metasilicate, then the resulting suspension was poured into a suspension of aluminosilicate raw material and stirred for 120 s in a mixer (TL-020, DzerzhinskTechnoMash, Dzerzhinsk, Russia) at 180 rpm. After stirring, aluminium powder was added to the geopolymer mixture and stirred for a further 60 s under the same conditions.

The resulting mixture was poured into silicone cube moulds with a rib length of 30 mm and sent for curing. One-stage low-temperature curing in a forced air convection oven (DO-80-01, Smolensk Special Design and Technology Bureau, Smolensk, Russia) at a temperature of 80 °C for 24 h was used as the temperature and time regime. Afterwards, the cured porous geopolymers were studied.

## 3. Results and Discussion

### 3.1. Physical and Mechanical Properties of Synthesised Porous Geopolymers Containing Metallurgical Slag

**Figure 4:**
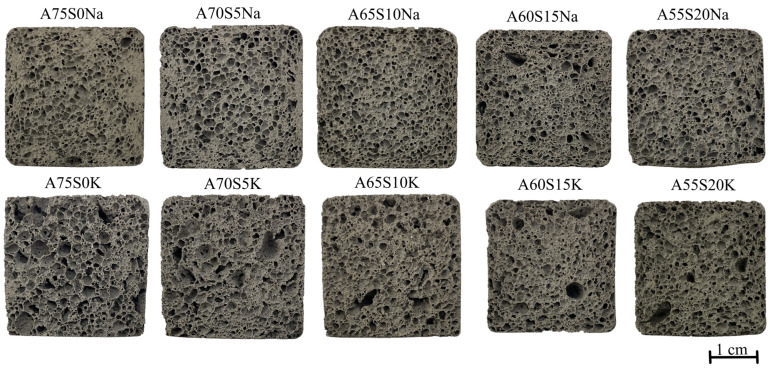
Macrostructure of synthesised samples containing metallurgical slag.

**Figure 5:**
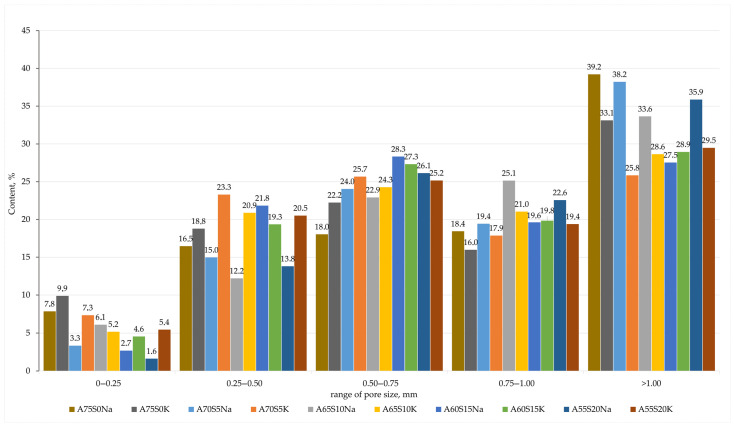
Averaged histograms of pore size ranges of compositions containing metallurgical slag.

Based on the sample investigation methods (Section 2.2) and the geopolymer synthesis methodology (Section 2.3), samples with internal structure and physical and mechanical properties were obtained, as shown in Figure 4 and Table 5. The average chemical oxide composition of the synthesised compositions is shown in Table 6. Figure 5 shows the histograms of the average pore size distribution in the studied samples.

When potassium containing an alkaline activator was used, the foaming intensity of the samples was very high, resulting in some samples (e.g., A60S15K and A55S20K) having an irregular porous structure with inclusions of large macropores up to 5 mm in diameter, as shown in Figure 4 and Figure 5. Furthermore, the high chemical activity of the potassium-containing alkali activator resulted in its intense interaction with aluminium powder. This resulted in the rapid release of gaseous hydrogen, a significant portion of which might have been lost, even at the stage of mixing the raw material charge. This uncontrollable process complicated the synthesis technology of the final material and also affected the density of the final samples, depending on the amount of gas lost. For instance, as illustrated in Table 5, the distribution of density values in samples was evidently influenced by the presence of potassium-containing alkaline activators. At the same time, the A75S0K and A65S10K samples had a more homogeneous porous structure and satisfactory strength properties due to the positive influence of the steelmaking slag.

The incorporation of 5% SS into the composition of geopolymers did not exert a significant effect on their properties; the set of physical and mechanical characteristics was commensurate with those observed in compositions A75S0Na and A75S0K. It should be noted that when the steelmaking slag content exceeded 10%, there was a deterioration in the physical and mechanical properties of all samples. This was probably due to the increase in the CaO content of the compositions by an average of 28%, which is clearly shown in Table 6.

Thus, based on the obtained results of the complex of physicochemical and mechanical investigations, it is revealed that the optimal samples were A75S0Na, A75S0K, A65S10Na, and A65S10K; in this respect, a further detailed study is presented.

### 3.2. Studies of the Thermal Transformations Occurring During the Firing of the Best Geopolymer Compositions with Metallurgical Slag Content

**Figure 6:**
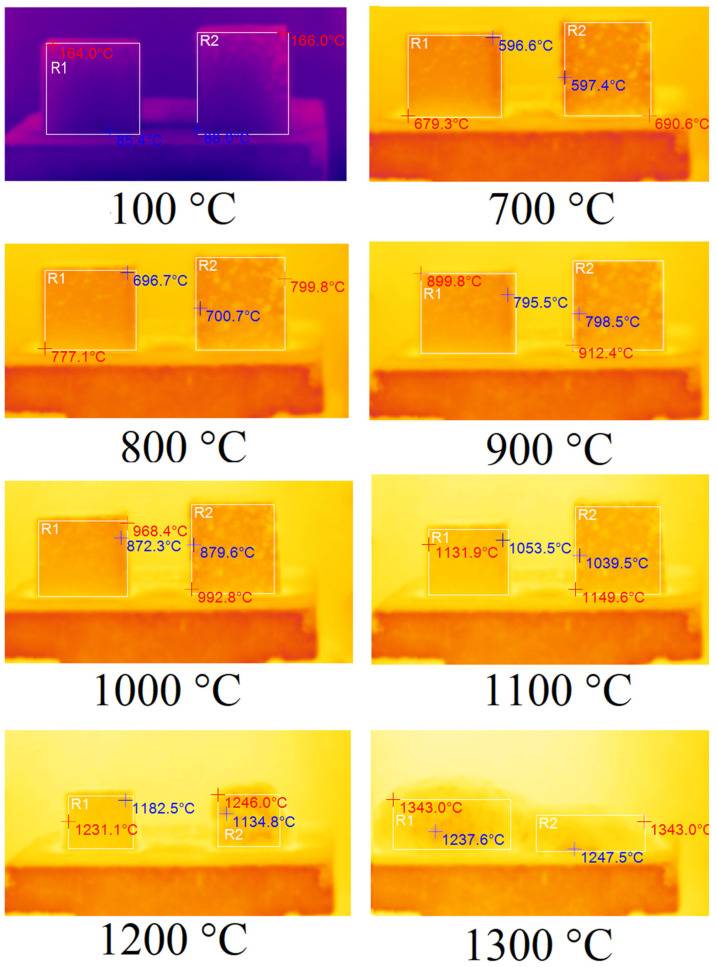
Thermal images of samples A75S0Na (**left**) and A65S10Na (**right**).

In order to determine the thermal properties of optimum samples of foamed geopolymer materials, they were fired in an electric chamber furnace TK.8.1300.N.1F (LLC ‘Thermoceramics’, Moscow, Russia) to a temperature of 1300 °C at a heating rate of 10 °C/min. The changes occurring to the samples during their heat treatment were recorded using a DT-9897H thermal imager (Shenzhen Everbest Machinery Industry Co., Ltd., Shenzhen, China) with a step of 100 °C fixed on a tripod. Tests of samples A75S0Na and A65S10Na using sodium alkali activator are shown in Figure 6. The temperature indicated in the figure caption was the programme temperature represented on the electronic furnace sensor.

In Figure 6, the corresponding samples are highlighted by areas R1 and R2. The maximum temperature of the sample is indicated in red, while the minimum temperature is indicated in blue. According to the results obtained, the samples A75S0Na and A65S10Na did not undergo any changes when heated from room temperature up to 700 °C. From 700 °C, the samples started to shrink due to sintering. From 900 °C, the specimens began to deform geometrically due to softening. In the 1100–1200 °C temperature range, significant melting of the A65S10Na sample was observed, whereas the A75S0Na sample did not undergo such a sharp deformation in the same temperature range. This factor allows us to conclude that the introduced steelmaking slag in the composition of geopolymers based on coal production waste had fluxing properties. At temperatures above 1200 °C, complete melting of the samples was observed. It is noteworthy that the melt of sample A75S0Na, which exhibits a higher viscosity compared to the melt of sample A65S10Na, undergoes foaming and an increase in volume due to chemical transformations occurring during high-temperature treatment.

**Figure 7:**
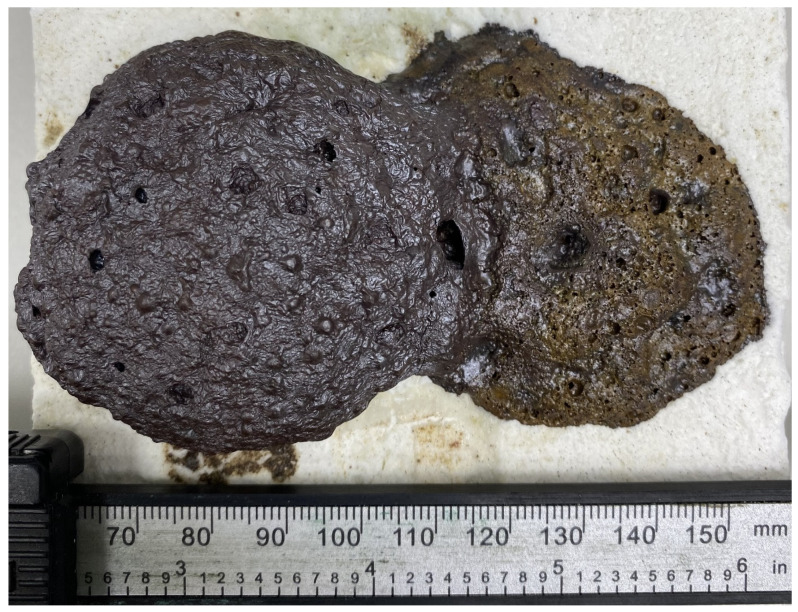
Appearance of samples A75S0Na[M] (**left**) and A65S10Na[M] (**right**) after thermal tests.

The fluxing properties of steelmaking slag were primarily related to the presence of a significant amount of calcium oxide CaO, which was capable of interacting at high temperatures with SiO_2_, and significant amounts of which were present in the ASM. As a result, the calcium silicates formed had higher reactivity and lower melting points than pure quartz. The appearance of the A75S0Na[M] and A65S10Na[M] samples after thermal treatment is shown in Figure 7.

As can be seen from Figure 7, samples A75S0Na[M] and A65S10Na[M], after thermal treatment, were glassy, with multiple gas inclusions, and had a reddish-brown colour due to the presence of mixed iron (II, III) oxide Fe_3_O_4_ in the samples. In addition, the colouring of the A65S10Na[M] sample was slightly different, apparently due to the oxidation of the metallophase in the steelmaking slag, which was predominantly expressed by iron with the formation of corresponding oxides.

**Figure 8:**
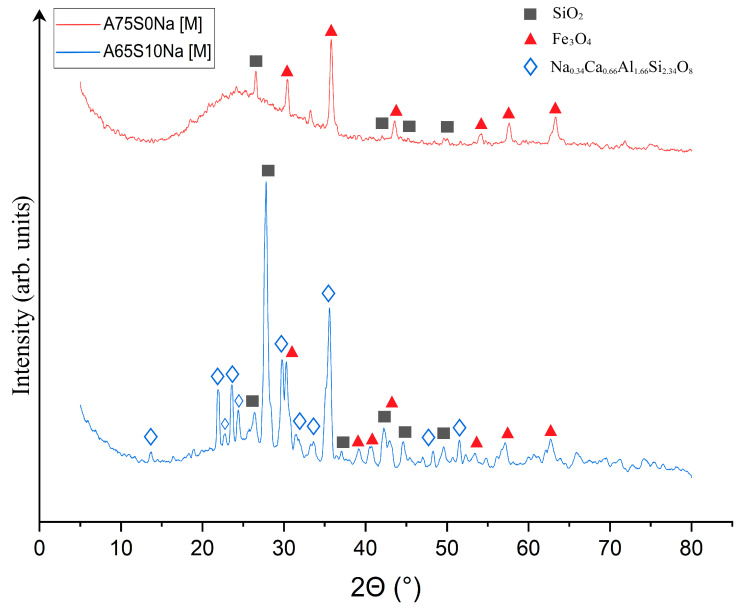
X-ray phase analysis of samples A75S0Na[M] and A65S10Na[M]: ▲—Fe_3_O_4_; ■—SiO_2_; ◊—Na_0.34_Ca_0.66_Al_1.66_Si_2.34_O_8_.

Qualitative X-ray phase analysis was then carried out on samples A75S0Na[M] and A65S10Na[M], and the results are shown in Figure 8.

As can be seen from Figure 8, heat treatment caused a change in the phase composition of the samples due to the chemical transformations that occurred during the process. It was found that the XRD of the A75S0Na[M] sample showed a decrease in the SiO_2_ peaks associated with its transition to the amorphous state, which was confirmed by the presence of a halo in the range 5–25°. The quantitative composition of sample A75S0Na consisted of a 97.1% ± 4.3% amorphous and 2.9% ± 0.3% crystalline structure, with 1.5% ± 0.2% Fe_3_O_4_ and 1.4% ± 0.1% SiO_2_. The appearance of a new phase—Fe_3_O_4_—formed by the oxidation of divalent iron compounds was present in the ASM. This process can be conventionally described by Equations (5) and (6):

4FeO + 3O_2_ = 2Fe_2_O_3_ + Fe (5)

3Fe + 2O_2_ = Fe_3_O_4_ (6)

It can be posited that the formation of Na_0.34_Ca_0_._66_Al_1.66_Si_2.34_O_8_ was associated with the high-temperature interaction of sodium silicates with calcium aluminates, as indicated by Equation (7):

0.17Na_2_O·2.34SiO_2_ + 0.66CaO·0.83Al_2_O_3_ = Na_0.34_Ca_0_._66_Al_1.66_Si_2.34_O_8_ (7)

In general, it can be seen that geopolymeric materials based on the Novocherkasskaya SDPP ash-slag mixture did not undergo strong transformations as a result of thermal treatment, which was associated with a certain inertness of the used aluminosilicate raw materials.

The XRD of the A65S10Na[M] sample also showed a decrease in the peaks of the SiO_2_ phase and the appearance of a new phase of mixed-iron (II,III) oxide Fe_3_O_4_, whose formation is described above. In addition, there was an appearance of several peaks represented by a crystalline phase in the form of Na-anorthite (sodium–calcium aluminosilicate) of complex composition with the formula Na_0.34_Ca_0.66_Al_1.66_Si_2.34_O_8_. The formation of this phase can be explained by a complex multistage mechanism, whereby the active form of calcium oxide contained in the steelmaking slag interacted with quartz contained in the ASM to form reactive calcium silicates of variable compositions. The calcium silicates formed in turn interacted with sodium-containing components introduced by the alkaline activator, as well as aluminium-containing compounds in the raw material, to form a new phase. The quantitative XRD of sample A65S10Na consisted of an 87.9 ± 2.8% amorphous and 12.1 ± 0.7% crystalline structure (5.1 ± 0.3% Fe_3_O_4_; 3.4 ± 0.2% SiO_2_, 3.6 ± 0.2% Na_0.34_Ca_0.66_Al_1.66_Si_2.34_O_8_).

**Figure 9:**
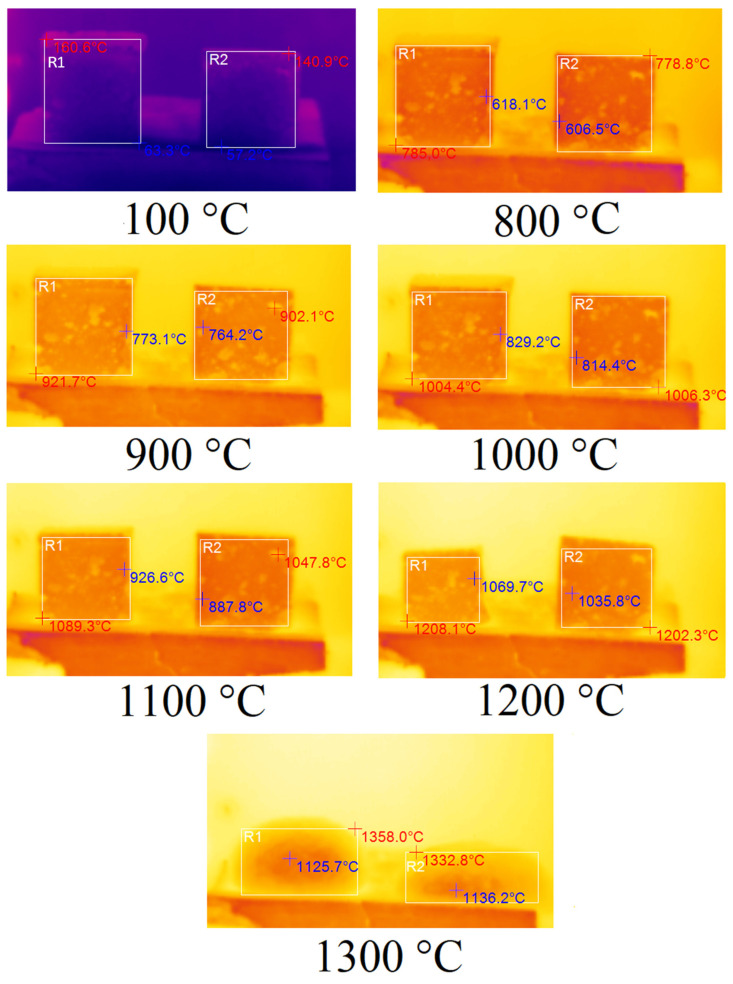
Thermal images of samples A75S0K and A65S10K.

Tests of samples A75S0K and A65S10K using a potassium alkaline activator are shown in Figure 9.

As mentioned earlier, in Figure 9, the corresponding samples are highlighted by the areas R1 and R2. The maximum temperature of the sample is indicated in red, while the minimum temperature is indicated in blue. As demonstrated in Figure 9, no observable changes were evident in samples A75S0K and A65S10K when heated from room temperature to 800 °C. However, commencing at 800 °C, a linear shrinkage process was initiated in the samples due to their sintering. Additionally, sample A75S0K, which was devoid of steelmaking slag, underwent a greater degree of shrinkage compared to sample A65S10K. At 1200 °C, both samples exhibited geometric deformation due to their softening, with the deformation of sample A75S0K being more pronounced. However, when the temperature reached 1200 °C, there was an abrupt melting of the A65S10K sample, which was clearly observed at 1300 °C. A75S0K and A65S10K specimens exhibited a higher degree of thermal resistance, as evidenced by their ability to withstand higher temperatures without undergoing any observable changes. This finding is consistent with the hypothesis that potassium-containing geopolymeric materials possess enhanced thermal resistance properties. Furthermore, the manufacturer of the liquid glasses asserts that the fire resistance of sodium liquid glass is up to 600 °C, and of potassium liquid glass, it is up to 900 °C. Consequently, compositions derived from distinct liquid glasses exhibited varied thermal resistance.

**Figure 10:**
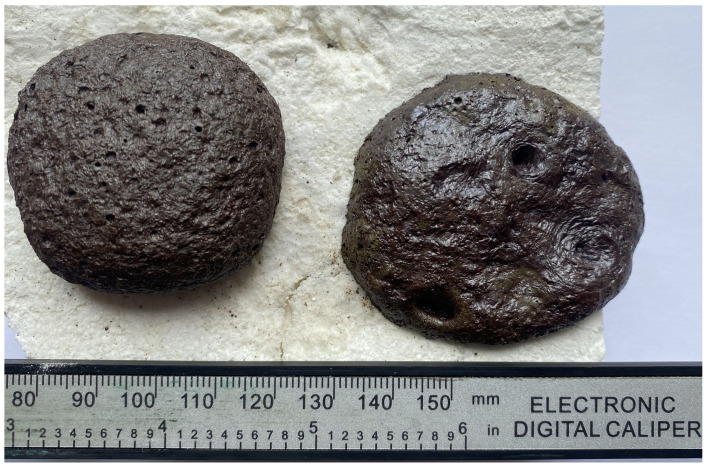
Appearance of samples A75S0K[M] and A65S10K[M] after thermal tests.

Thermal testing of the A75S0K and A65S10K samples resulted in the A75S0K[M] and A65S10K[M] samples, the appearance of which is shown in Figure 10.

**Figure 11:**
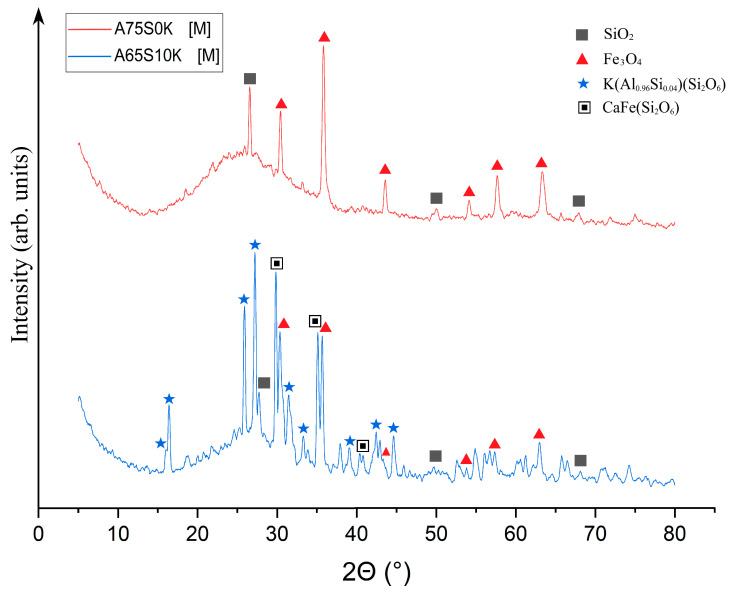
X-ray phase analysis of samples A75S0K[M] and A65S10K[M]: ▲—Fe_3_O_4_; ■—SiO_2_; ★—K(Al_0.96_Si_0.04_)(Si_2_O_6_); ▣—CaFe(Si_2_O_6_).

As demonstrated in Figure 10, post-thermal testing, samples A75S0K[M] and A65S10K[M] exhibited a glassy consistency, accompanied by numerous gas inclusions and a reddish-brown hue, attributable to the presence of mixed iron (II, III) oxide Fe_3_O_4_ within the samples. These outcomes bear a striking resemblance to those observed in the testing of samples A75S0Na and A65S10Na. The results of the qualitative X-ray phase analysis of the A75S0K[M] and A65S10K[M] samples are displayed in Figure 11.

As can be seen from Figure 11, a decrease in SiO_2_ peaks was observed in the XRD of the A75S0K[M] sample compared to the XRD of the original A75S0K sample. There were quartz changes to the amorphous form during heat treatment, which were confirmed by the presence of a halo in the range 5–25°. The quantitative XRD of sample A75S0K[M] consisted of a 98.3 ± 3.5% amorphous and 1.7 ± 0.2% crystalline structure (0.7 ± 0.1% Fe_3_O_4_; 1.0 ± 0.1% SiO_2_). The appearance of the Fe_3_O_4_ phase, whose formation mechanism is described above, was also observed. The XRD of sample A65S10K[M] also showed a decrease in SiO_2_ peaks compared to sample A65S10K and the appearance of an Fe_3_O_4_ phase. The quantitative XRD of sample A65S10K[M] consisted of a 79.2 ± 0.8% amorphous and 20.8 ± 0.9% crystalline structure (1.0 ± 0.1% Fe_3_O_4_; 1.1 ± 0.1% SiO_2_, 11.4 ± 0.3% K(Al_0.96_Si_0.04_)(Si_2_O_6_); 7.3 ± 0.4% CaFe(Si_2_O_6_)). In addition, a potassium-containing activator, including potassium silicates and potassium hydroxide, resulted in the formation of a complex phase of K(Al_0.96_Si_0.04_)(Si_2_O_6_)–leucite due to their interaction with quartz and aluminium-containing compounds in the feedstock. In nature, it is a high-temperature rock-forming material of magmatic origin, formed in potassium-rich sub-volcanic rocks. It is hypothesised that the formation of this phase occurs during the interaction of potassium silicates with aluminium silicate intermediates, as depicted in Equation (8):

K_2_O · 4SiO_2_ + 0.96Al_2_O_3_ · 0.08SiO_2_ = 2K(Al_0.96_Si_0.04_)(Si_2_O_6_) (8)

Also observed was the appearance of the CaFe(Si_2_O_6_) phase—hedenbergite—which is an iron-calcium silicate formed by the interaction of quartz SiO_2_ with free calcium oxide present in steelmaking slag. This reaction produces reactive calcium silicates that interact with iron oxides to form the observed phase. This process is delineated in Equation (9):

CaO · 2SiO_2_ + FeO = CaFe(Si_2_O_6_) (9)

It has been established that the incorporation of steelmaking slag into the composition of geopolymeric materials derived from coal power plant waste contributes to a reduction in their melting temperature and decreases the viscosity of the melt. This is attributable to the fluxing properties of the free calcium oxide present in the steelmaking slag, which in turn reduces the thermal properties of the geopolymeric material. Notwithstanding, the utilisation of steelmaking slag holds potential for the synthesis of high-temperature materials based on ash-slag blends, a process attributable to its capacity to reduce the melting point of the initial blend. In addition, it has been determined that the incorporation of potassium-based alkaline activators enhances the thermal stability of geopolymer materials, thereby increasing the softening point of the material by an average of 100 °C in comparison with conventionally synthesised geopolymers that employ sodium-based alkaline activators.

### 3.3. Physical and Mechanical Properties of Synthesised Porous Geopolymers Containing Drilling Sludge

**Figure 12:**
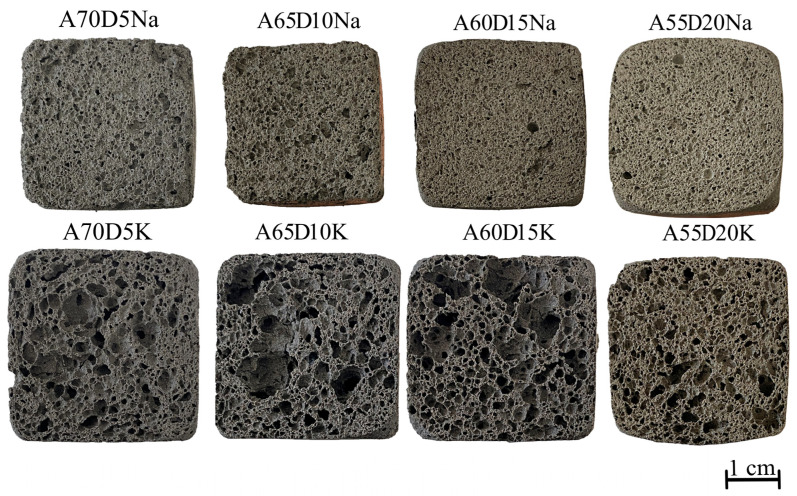
Macrostructure of synthesised samples containing drilling sludge.

**Figure 13:**
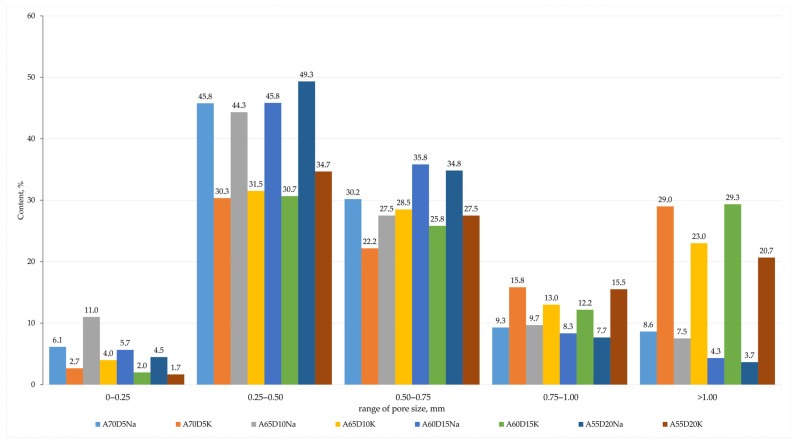
Averaged histograms of pore size ranges of compositions containing drilling sludge.

Based on the methods of sample investigation (Section 2.2) and the methodology of geopolymer synthesis (Section 2.3), samples with an internal structure and physical and mechanical properties were obtained, which are presented in Figure 12 and Table 7. The averaged chemical oxide composition of the synthesised compositions is shown in Table 8. Figure 13 shows the histograms of the average pore size distribution in the studied samples.

In summary, the incorporation of DS in quantities ranging from 5 to 20% has been demonstrated to result in a deterioration of the physical and mechanical properties of porous geopolymers. The addition of 20% DS was found to reduce the strength of geopolymer samples by 17% from 1.54 to 1.29 MPa compared to the use of a 10% steelmaking slag additive. The increase in density of the samples was probably due to the presence of surfactants in drilling sludge, which acted as defoamers. These surfactants are incorporated into drilling fluids used in the drilling of plastic rock to prevent cuttings particles from adhering to the drill bit and to each other, i.e., to prevent cuttings dispersion. Furthermore, the enhanced compressive strength exhibited by samples containing SS may be attributed to their pozzolanic properties, a hypothesis that is substantiated by X-ray phase analysis, which revealed the presence of calcium aluminate phases, analogous to those observed in Portland cement. Furthermore, the chloride content of these samples may induce a corrosive effect on metal structures. The effect of chlorides on steel structures can be explained as follows. Cl- ions have been observed to adsorb defects in FeOOH, which is known as the passivation layer on the surface of steel. Subsequently, Na+ (or K+) ions are induced to form Cl-Na(K) bonds. The bonds formed due to vibrations weaken the interaction between the layers of the passivation film, leading to its destruction. Consequently, external influences result in further oxidation of the metal [57]. In this regard, further research into the production of geopolymers containing drilling sludge requires the development of a technology for removing chloride-containing impurities from them. The removal of sodium and potassium chlorides, which possess high solubility, from the drilling sludge can be achieved through a thorough washing process.

While the compressive strength values obtained from specimens containing 20% drilling sludge were marginally lower than those obtained from specimens containing 15% metallurgical slag, specimens A55D20Na and A55D20K demonstrated an optimal performance.

### 3.4. Investigation of Thermal Transformations During Firing of the Best Geopolymer Compositions Containing Drilling Sludge

**Figure 14:**
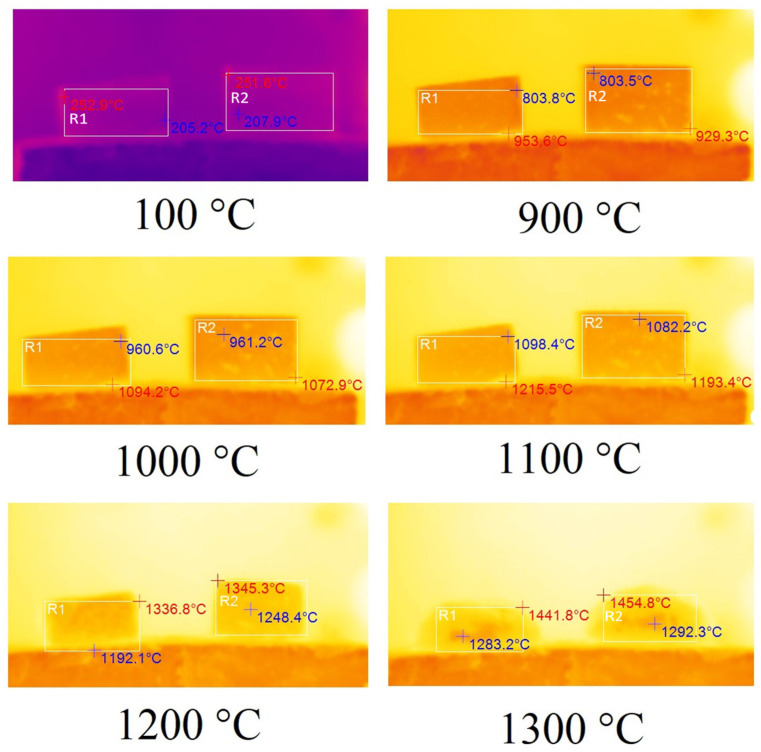
Thermal images of samples A75D20Na (**left**) and A65D20K (**right**).

Following the methodology described above, the studies of thermal transformations during the combustion of geopolymers containing 20% drilling sludge were carried out separately. The results of these studies are presented in Figure 14.

As demonstrated in Figure 14, samples A65D20Na and A65D20K demonstrated no alteration when subjected to heating from room temperature to 900 °C. Beginning at 900 °C, the samples underwent a linear shrinkage process due to their sintering. It is noteworthy that at a temperature of 1200 °C, the melting process of the samples studied began, and at 1300 °C, the formation of a foamed melt occurred. However, it can be clearly seen that the amount of foam formed was less than when melting samples containing steelmaking slag or no additional additives. Consequently, it can be deduced that the incorporation of drilling sludge served to mitigate foaming during the melting process of geopolymer samples.

**Figure 15:**
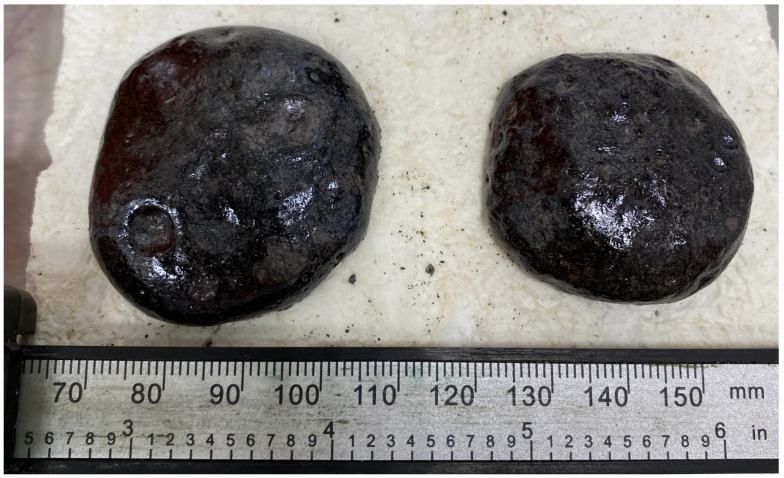
Appearance of A75D20Na[M] and A65D20K[M] samples after thermal testing.

The appearance of heat-treated samples is shown in Figure 15.

**Figure 16:**
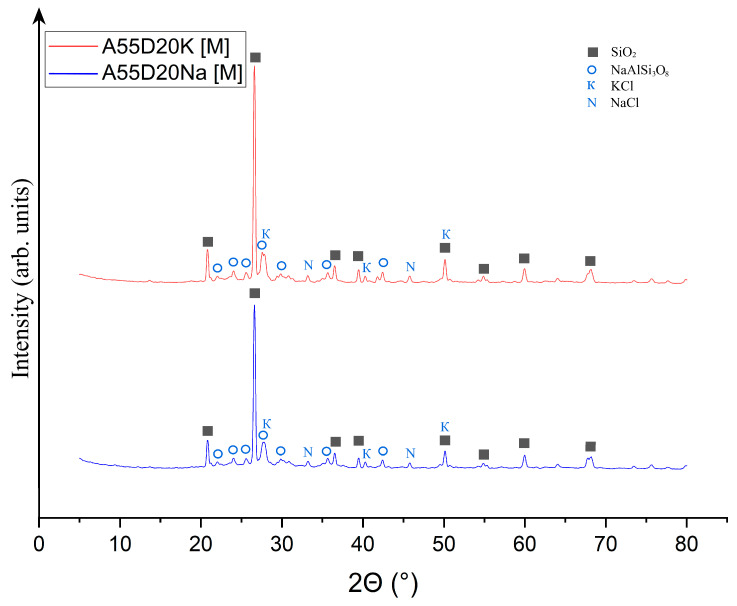
X-ray phase analysis of samples A75D20Na[M] and A65D20K[M]: ■—SiO_2_; K—KCl; N—NaCl; **◯**—NaAlSi_3_O_8_.

As demonstrated in Figure 15, the appearance of the obtained samples differed slightly from that of the geopolymer samples devoid of additives and those containing steelmaking slag additives. In this case, the formation of a vitrified, smooth surface, almost black in colour, was observed on the surface of the sample. The results of the qualitative X-ray phase analysis of samples A75D20Na[M] and A65D20K[M] are presented in Figure 16.

As demonstrated in Figure 16, the phase composition of the A55D20Na[M] and A55D20K[M] samples was found to be identical. In this regard, it can be concluded that the chemical nature of the alkaline activator did not have any effect on phase formation during thermal treatment of geopolymers with the addition of drilling sludge. It is evident that the phases present, expressed by quartz, sylvin (potassium chloride), halite (sodium chloride), and albite, were also observed in the feedstock, which consisted of ASM and DS. Nevertheless, a decrease in the intensity of quartz peaks and a halo formation was observed, indicating its partial transition to the amorphous phase. The quantitative XRD of sample A55D20Na[M] consisted of a 39.1 ± 1.3% amorphous and 60.9 ± 1.1% crystalline structure (32.5 ± 0.6% SiO_2_; 27.9 ± 0.5% NaAlSi_3_O_8_; 0.3 ± 0.0% KCl; 0.2 ± 0.0% NaCl). The quantitative XRD of sample A55D20K[M] consisted of a 28.1 ± 0.2% amorphous and 71.9 ± 1.2% crystalline structure (41.9 ± 0.8% SiO_2_; 29.7 ± 0.4% NaAlSi_3_O_8_; 0.2 ± 0.0% KCl; 0.1 ± 0.0% NaCl). Consequently, no substantial phase transitions occurred during the thermal treatment of these samples.

### 3.5. This Study Will Compare the Comparative Characteristics of Sample A65S10Na with Those of Other Samples of Porous Geopolymer Materials

As demonstrated by the preceding studies, sample A65S10Na was identified as the optimal specimen, exhibiting the lowest density and the highest strength. A comparative analysis was performed of the primary key indicators of sample A65S10Na with the studies of Tsousi et al. [54], who synthesised a porous geopolymer sample based on copper slag using aluminium powder (0.15%) as a foaming agent. A comparison was also made with the research of Liang et al. [55], who synthesised a porous geopolymer sample based on 80% ground granulated blast furnace slag (GGBS) and 20% rice husk ash (RHA) using a hydrogen peroxide solution (2%) as a foaming agent. The results of the comparative analysis are presented in Table 9.

As demonstrated in Table 9, sample A65S10Na exhibited the optimal set of properties in comparison to the other samples. The copper slag utilised by Tsousi et al. [54] in the production of geopolymers exhibited an absence of binding properties, which was purportedly the underlying cause of the diminished strength of the sample based on it at a higher density. The authors’ incorporation of aluminium powder (0.15%) was instrumental in achieving the elevated density of the specimen. The elevated compressive strength exhibited by the sample synthesised by Liang et al. [55] can be attributed to its substantial density, as well as the considerable proportion of granulated ground blast furnace slag (GGBS), which possesses pronounced binding properties and is extensively utilised in the cement industry. The samples were cured at an ambient temperature for a period of 28 days. In this case, the samples cured at 80 °C for 24 h, as performed in this study, may be more economically viable. Furthermore, the GGBS employed by Liang et al. [55] contained double the amount of SiO_2_ (32.5%) compared to the steel slag utilised in this study (14.7%), which may have consequently exerted an effect on the degree of geopolymerisation. Notwithstanding, the compressive strength and density ratios of sample A65S10Na are satisfactory for its use as a thermal insulation material, since the compressive strength requirements range from 0.5 to 2.5 MPa. In general, the thermal conductivity values of all three materials under consideration are logically dependent. The density of the substance in question is a pivotal factor in determining its capacity to facilitate the desired outcome. However, the most compelling of these is sample A65S10Na, which exhibited an almost twofold reduction in thermal conductivity relative to the Liang et al. [55] sample and a 1.23-fold decrease compared to the Tsousi et al. [54] sample.

## 4. Conclusions

A thorough investigation was thus conducted into the impact of steelmaking slag, drilling sludge, and alkaline activators of varying chemical composition on the characteristics of foamed geopolymer materials derived from an ash and slag mixture. The study yielded the following findings:

1. An increase in the content of steelmaking slag by more than 10% was shown to result in a deterioration of the physical and mechanical properties of the samples. This deterioration was characterised by a decrease in density and compressive strength, with an average reduction of 8% and 20%, respectively. This phenomenon can be attributed to the elevated levels of free CaO present in the slag, a decline in SiO_2_ and Al_2_O_3_ content within the porous geopolymer, and an augmentation in the chemical activity of the geopolymer mixture resulting from the presence of pozzolanic phases in the slag.
2. The investigation of the thermal properties of the optimum samples indicated that steelmaking slag exerts a fluxing effect on the properties of foamed geopolymer materials. This phenomenon can be attributed to the presence of calcium silicates, which are capable of being readily fused in the synthesised samples.
3. The incorporation of 20% drilling sludge resulted in a 27% reduction in the compressive strength of geopolymer samples in comparison to the utilisation of an additive in the form of 10% steelmaking slag, since drilling sludge does not possess pozzolanic properties.
4. The addition of drilling sludge to geopolymers, in quantities ranging from 5 to 20% of the total mixture, was shown to reduce foam formation in the melt. Furthermore, it was observed that this addition did not result in significant phase changes during the heat treatment process, with the exception of the transition of crystalline quartz phases to an amorphous state.
5. A series of experiments was conducted to ascertain the optimal sample. It was determined that the optimal sample was A65S10Na, which consisted of 65% ASM, 10% SS, 2.5% NaOH, 22.5% sodium metasilicate, 5% water, and 2% aluminium powder.

Consequently, based on the obtained results, further research directions can be determined. The primary objectives of future investigations are threefold: to study the processes of removing chlorides from drilling sludge, to optimise the technology of removing chlorides from drilling sludge, and to study the water-absorbing properties of porous geopolymer materials depending on their composition and physical and mechanical properties.

## Figures and Tables

**Table 1:** Average chemical composition of raw materials, wt.%.

Component	SiO_2_	Al_2_O_3_	Fe_2_O_3_	MgO	Na_2_O	K_2_O	CaO	TiO_2_	MnO	P_2_O_5_	SO_3_	Cl	LOI
ASM	52.1	18.6	10.1	1.9	0.9	2.8	3.2	0.7	0.1	0.1	0.3	–	9.2
SS	14.7	15.6	–	5.7	–	0.1	49.0	0.3	–	0.2	3.2	–	−11.6 *
DS	47.4	11.0	4.3	1.4	2.6	6.3	7.6	0.5	0.1	0.2	0.4	5.0	13.2
Sodium metasilicate	33.6	–	–	–	11.2	–	–	–	–	–	–	–	55.2
Potassium metasilicate	35.7	–	–	–	–	11.6	–	–	–	–	–	–	52.7

* The deciphering of the SS LOI is presented in Table 3.

**Table 2:** Physical characteristics of raw materials.

Material	True Density, kg/m^3^	Bulk Density, kg/m^3^	Porosity, %	Particle Size, µm		Specific Surface Area, m^2^/kg
				D50	D99	
ASM	2364 ± 75	1059 ± 47	55.2 ± 1.6	23.23 ± 1.03	57.97 ± 2.20	191 ± 8
SS	3145 ± 112	680 ± 24	78.4 ± 2.0	12.34 ± 0.54	27.12 ± 1.00	270 ± 11
DS	2452 ± 90	827 ± 30	66.3 ± 2.9	10.16 ± 0.41	23.42 ± 0.76	421 ± 10

**Table 3:** Elemental composition of the metallophase contained in SS.

Component	Fe	Mn	Mo	Nb	Cr	Cu
* LOI SS	9.70	1.50	0.30	<0.01	0.06	0.04

* Component of the SS LOI is presented in Table 1.

**Table 4:** Component composition of the raw material mixture, wt.%.

Composition	ASM	SS	DS	NaOH	KOH	Sodium Metasilicate	Potassium Metasilicate	Water, Over 100	Aluminium Powder, Over 100
A750Na	75.0	–	–	2.5	–	22.5	–	5.0	2.0
A750K	75.0	–	–	–	2.5	–	22.5	5.0	2.0
A70S5Na	70.0	5.0	–	2.5	–	22.5	–	5.0	2.0
A70D5Na	70.0	–	5.0	2.5	–	22.5	–	5.0	2.0
A70S5K	70.0	5.0	–	–	2.5	–	22.5	5.0	2.0
A70D5K	70.0	–	5.0	–	2.5	–	22.5	5.0	2.0
A65S10Na	65.0	10.0	–	2.5	–	22.5	–	5.0	2.0
A65D10Na	65.0	–	10.0	2.5	–	22.5	–	5.0	2.0
A65S10K	65.0	10.0	–	–	2.5	–	22.5	5.0	2.0
A65D10K	65.0	–	10.0	–	2.5	–	22.5	5.0	2.0
A60S15Na	60.0	15.0	–	2.5	–	22.5	–	5.0	2.0
A60D15Na	60.0	–	15.0	2.5	–	22.5	–	5.0	2.0
A60S15K	60.0	15.0	–	–	2.5	–	22.5	5.0	2.0
A60D15K	60.0	–	15.0	–	2.5	–	22.5	5.0	2.0
A55S20Na	55.0	20.0	–	2.5	–	22.5	–	5.0	2.0
A55D20Na	55.0	–	20.0	2.5	–	22.5	–	5.0	2.0
A55S20K	55.0	20.0	–	–	2.5	–	22.5	5.0	2.0
A55D20K	55.0	–	20.0	–	2.5	–	22.5	5.0	2.0

**Table 5:** Averaged physical and mechanical characteristics of porous geopolymers with metallurgical slag.

Composition	Density, kg/m^3^	Compressive Strength, MPa	Porosity, %	Thermal Conductivity, W/(m·K)
A75S0Na	356 ± 7	1.46 ± 0.06	84.7 ±0.3	0.0793 ± 0.0014
A75S0K	338 ± 13	1.39 ± 0.05	85.5 ±0.6	0.0754 ± 0.0028
A70S5Na	346 ± 8	1.42 ± 0.06	85.3 ±0.7	0.0742 ± 0.0022
A70S5K	351 ± 11	1.38 ± 0.07	84.5 ±0.9	0.0777 ± 0.0034
A65S10Na	311 ± 15	1.54 ± 0.06	86.7 ±0.7	0.0698 ± 0.0032
A65S10K	363 ± 34	1.39 ± 0.06	84.4 ±1.4	0.0808 ± 0.0071
A60S15Na	347 ± 13	1.37 ± 0.04	85.1 ±0.6	0.0773 ± 0.0027
A60S15K	372 ± 14	1.34 ± 0.03	84.0 ±0.6	0.0828 ± 0.0030
A55S20Na	323 ± 9	1.33 ± 0.06	86.1 ±0.4	0.0723 ± 0.0018
A55S20K	328 ± 19	1.25 ± 0.04	85.9 ±0.8	0.0733 ± 0.0040

**Table 6:** Average chemical composition of synthesised porous geopolymer with metallurgical slag content, wt.%.

Composition	SiO_2_	Al_2_O_3_	Fe_2_O_3_	MgO	Na_2_O	K_2_O	CaO	TiO_2_	MnO	P_2_O_5_	SO_3_	LOI
A75S0Na	46.6	14.0	7.6	1.4	5.1	2.1	2.4	0.5	0.1	0.1	0.2	19.9
A75S0K	47.1	14.0	7.6	1.4	0.7	6.6	2.4	0.5	0.1	0.1	0.2	19.3
A70S5Na	45.3	13.9	7.2	1.6	5.1	2.0	4.7	0.5	0.1	0.1	0.4	19.1
A70S5K	45.8	14.0	7.1	1.5	0.6	6.9	4.5	0.5	0.1	0.1	0.4	18.5
A65S10Na	42.9	13.7	7.5	1.8	5.0	1.8	7.1	0.5	0.2	0.1	0.5	18.9
A65S10K	43.4	13.7	7.5	1.8	0.6	6.4	7.0	0.5	0.2	0.1	0.5	18.3
A60S15Na	41.1	13.5	7.4	2.0	5.0	1.7	9.4	0.5	0.3	0.1	0.7	18.3
A60S15K	41.5	13.5	7.4	2.0	0.5	6.2	9.5	0.5	0.3	0.1	0.7	17.8
A55S20Na	39.2	13.4	7.5	2.2	5.0	1.6	11.7	0.4	0.3	0.1	0.8	17.8
A55S20K	40.8	13.8	7.6	2.3	0.5	4.3	12.0	0.4	0.3	0.1	0.8	17.1

**Table 7:** Average physicochemical characteristics of porous geopolymers with drilling sludge content.

Composition	Density, kg/m^3^	Compressive Strength, MPa	Porosity, %	Thermal Conductivity, W/(m·K)
A70D5Na	435 ± 21	1.29 ± 0.03	80.89 ± 0.34	0.0966 ± 0.0043
A70D5K	344 ± 15	0.96 ± 0.04	84.80 ± 0.51	0.0767 ± 0.0028
A65D10Na	520 ± 18	1.22 ± 0.02	77.89 ±0.75	0.1149 ± 0.0039
A65D10K	354 ± 14	0.75 ± 0.01	84.95 ±0.62	0.0787 ± 0.0031
A60D15Na	536 ± 56	1.19 ± 0.04	77.21 ±2.40	0.1184 ± 0.0126
A60D15K	389 ± 5	0.79 ± 0.01	78.77 ±0.25	0.0895 ± 0.0010
A55D20Na	532 ± 33	1.29 ± 0.02	77.37 ±1.40	0.1176 ± 0.0073
A55D20K	426 ± 24	0.95 ± 0.01	81.88 ±1.01	0.0942 ± 0.0052

**Table 8:** Average chemical composition of synthesised porous geopolymer using drilling sludge, wt.%.

Composition	SiO_2_	Al_2_O_3_	Fe_2_O_3_	MgO	Na_2_O	K_2_O	CaO	TiO_2_	MnO	P_2_O_5_	SO_3_	Cl	LOI
A70D5Na	47.3	13.4	7.1	1.5	5.4	2.6	0.5	0.1	0.1	0.2	0.6	0.4	20.8
A70D5K	47.7	13.5	7.2	1.4	2.9	5.2	0.5	0.1	0.1	0.2	0.5	0.5	20.2
A65D10Na	47.0	13.1	6.9	1.3	5.6	2.7	0.5	0.1	0.1	0.2	0.7	0.8	21.0
A65D10K	47.5	13.3	6.6	1.4	3.0	5.4	0.5	0.1	0.1	0.2	0.8	0.7	20.4
A60D15Na	46.9	12.7	6.5	1.3	5.6	2.9	0.5	0.1	0.1	0.2	1.0	1.0	21.2
A60D15K	47.4	13.0	6.2	1.3	3.1	5.6	0.5	0.1	0.1	0.2	1.0	0.9	20.6
A55D20Na	47.3	13.4	7.1	1.5	5.4	2.6	0.5	0.1	0.1	0.2	0.6	0.4	20.8
A55D20K	47.7	13.5	7.2	1.4	2.9	5.2	0.5	0.1	0.1	0.2	0.5	0.5	20.2

**Table 9:** Comparative characteristics of porous geopolymer samples.

Composition	Density, kg/m^3^	Compressive Strength, MPa	Thermal Conductivity, W/(m·K)
A65S10Na	311	1.54	0.069
[54]	805	1.28	0.085
[55]	827	10.5	0.133

## Data Availability

The original contributions presented in this study are included in the article. Further inquiries can be directed to the corresponding author.

## Abbreviations

The following abbreviations are used in this manuscript:

ISW	Industrial solid waste
EIA	Energy Information Administration
ASM	Ash and slag mixture
SS	Steel slag
DS	Drilling sludge
